# Matrix Remodeling Associated 7 Deficiency Alleviates Carbon Tetrachloride-Induced Acute Liver Injury in Mice

**DOI:** 10.3389/fimmu.2018.00773

**Published:** 2018-04-18

**Authors:** Dandan Lin, Zhenjiang Sun, Ziqi Jin, Lei Lei, Yonghao Liu, Bo Hu, Benfang Wang, Ying Shen, Yiqiang Wang

**Affiliations:** ^1^Key Laboratory of Thrombosis and Hemostasis Ministry of Health, Collaborative Innovation Center of Hematology, Jiangsu Institute of Hematology, The First Affiliated Hospital of Soochow University, Medical College, Soochow University, Suzhou, China; ^2^Department of Hematology, Institute of Hematopoietic Stem Cell Transplantation, Collaborative Innovation Center of Hematology, The First Affiliated Hospital of Soochow University, Medical College, Soochow University, Suzhou, China

**Keywords:** matrix remodeling associated 7, acute liver injury, neutrophils, extracellular matrix, pro-inflammatory cytokines

## Abstract

Matrix remodeling associated 7 (MXRA7) was first noted to co-express with a group of matrix remodeling related genes, and its biological functions had remained unclear. In this study, we investigated the presumed function of MXRA7 in a carbon tetrachloride (CCl_4_)-induced acute liver injury model in mice. Wild-type, MXRA7^−/−^ mice, and mice that were pulsed with hydrodynamic injection of vehicle or MXRA7-harboring plasmids were challenged with a single dose of CCl_4_ for injury induction. The sera, spleens, and livers were harvested from mice for assay of cytokines/chemokines expression, cellular responses, or histological features. We found that MXRA7 deficiency alleviated, and MXRA7 overexpression aggravated liver damage in CCl_4_-challenged mice. FACS analysis showed that MXRA7 deficiency reduced the recruitment of neutrophils through downregulation the expression of CXCL1 and CXCL2 in liver, decreased the number of CD8^+^ T cells in liver and spleen, suppressed the release of IFNγ and TNFα from T cells, and decreased IFNγ in serum and liver. Western blot assay demonstrated that MXRA7 deficiency suppressed the activation of MAPK pathway and AKT/NF-κB pathway, respectively. Lastly, MXRA7 deficiency or overexpression regulated the expression of two matrix remodeling-related genes (fibronectin and TIMP1) in the liver. We concluded that MXRA7 was an active player in CCl_4_-induced liver injury, hypothetically by mediating the inflammation or immune compartments and matrix remodeling processes. Further exploration of MXRA7 as a possible new therapeutic target for management of inflammation-mediated liver injury was discussed.

## Introduction

Liver is a vital organ which performs important functions like extensive synthesis, retinoid storage, metabolism, detoxification, secretion of proteins, etc. (1, 2). Liver diseases are one panel of the endangering problems which lead to mortality and morbidity over the world (3, 4). Liver injuries or diseases can be caused by many factors, including drugs, toxins, alcohol, and virus infection (5, 6). Acute liver injury (ALI) occurs within a short period and is a common pathway to many liver diseases. The pathogenesis of ALI involves oxidative stress, hepatocyte apoptosis and necrosis, immune responses, etc. (6–8). ALI also involves inflammation and may progress to chronic liver injury, hepatic fibrosis, or even hepatocellular carcinoma (9). Therefore, searching for new therapeutic options for treatment of liver injury is critical for handling liver diseases in clinical practice.

In laboratory studies concerning liver injury, carbon tetrachloride (CCl_4_) is commonly used as a chemical toxin to induce ALI model in mice (10). CCl_4_ is metabolized by cytochrome P450 (CYP2E1) in liver to form trichloromethyl and trichloromethyl peroxy radicals, which in turn induce oxidative stress, lipid peroxidation, and hepatic injury (11). Except for oxidative stress, inflammation is another important mechanism mediating CCl_4_-induced liver injury (12, 13). In this process, pro-inflammatory cytokines and chemokines are crucial players that incur cell death and liver injury (14–16). Correspondingly, previous studies sug-gested that the liver injury can be prevented by suppressing oxidative stress and inflammation (17, 18). Therefore, metabolism and inflammatory response are thought to be therapy targets in the treatment of liver injury. On the other side, liver injury and recovery also involve matrix remodeling, a process that underlies structural changes occurred in any tissues, including liver (19, 20). Thus, dissecting the relationship between matrix remodeling and liver injury also provides new possibilities for developing novel therapy of liver injury. In a previous study, we sighted expression of a novel gene, matrix remodeling associated 7 (MXRA7), in liver at mRNA level (21), but its possible involvement in any physiological or pathological processes of liver had not been addressed in any study. MXRA7, first named in a bioinformatics study in 2002, belongs to the MXRA family consisting of eight genes (MXRA1–MXRA8) possibly involved in cell adhesion and matrix remodeling (22). Studies have reported that some members of this family are related with a variety of critical physiological and pathological processes, such as MXRA2 in matrix degradation (23), MXRA3 in actin polymerization and cell motility (24), MXRA5 in anti-inflammatory and anti-fibrotic responses (25), and MXRA6 in myofibroblast differentiation and extracellular matrix formation (26). However, the function of MXRA7 has been barely investigated except for our previous study showing a dynamic change of MXRA7 mRNA in inflammatory corneal diseases models in adult mice (21).

In the present study, we used mice genetically deficient of MXRA7 (MXRA7^−/−^) or artificially overexpressing MXRA7 to investigate the hypothetical role of MXRA7 in CCl_4_-induced ALI in mice. The levels of serum alanine aminotransferase (ALT) and aspartate aminotransferase (AST) and histopathological analysis were used to evaluate liver functions. The inflammatory response and pro-inflammatory cytokines as well as extracellular matrix molecules were measured to elucidate the underlying mechanism. Our findings indicated an important role of MXRA7 in liver injury *in vivo*.

## Materials and Methods

### Animals

Heterozygous MXRA7-deficient (MXRA7^+/−^) mice of both sexes on C57BL/6N background were purchased from the Medical Research Council (MRC, Swindon, UK). The MXRA7^−/−^ and wild-type (WT) breeders were generated by cross-breeding female and male MXRA7^+/−^ mice, and genotyped according to the protocol provided by MRC (Figure S1 in Supplementary Material). The breeders were used for generating WT and MXRA7^−/−^ colonies, respectively. The male mice of WT and MXRA7^−/−^ (aged 6–8 weeks) were used in the study. Specific pathogen-free male C57BL/6N mice (aged 6–8 weeks) were obtained from Nanjing Biomedical Research Institute of Nanjing University (Nanjing, China). All mice were housed in specific pathogen-free facility in Soochow University.

### MXRA7-Delivery Plasmid Construction and Hydrodynamic Gene Transfer (HGT) *In Vivo*

The murine MXRA7 cDNA was cloned from splenocytes of mouse and inserted into a minicircle (MC) plasmid (pMC.EF1, System Biosciences, Mountain View, CA, USA) to construct the MXRA7-delivery plasmid (pMC-MXRA7), while the mock pMC.EF1 plasmid was used as control plasmid in the study. Confirmation with sequencing, preparation, and purification of both plasmids were performed as previously described (27). pMC plasmids were delivered into mice by HGT technique (80 µg plasmids in 2.5 ml PBS/mouse). Three days postinjection of plasmids, the mice were subjected to ALI induction as described below.

### Acute Liver Injury

The animal experiments were divided into two sections, namely, the MXRA7 deficiency section using WT and MXRA7^−/−^ mice, and the MXRA7 overexpression section using mice challenged with pMC.EF1 (control) or pMC-MXRA7 plasmids. CCl_4_ (Sinopharm, Shanghai, China) was used to induce liver injury of differently severity in mice. For survival experiments, the mice were injected intraperitoneally (i.p.) with 6 ml/kg body weight CCl_4_ (1:1 dilution in corn oil) and the survival of mice were observed every day. Pilot experiments and previous studies had determined this lethal dose within 1 week in WT mice to be around 6 ml/kg body weight CCl_4_ (1:1 corn oil) (27). For short-term ALI induction and acute phase assay, the mice were injected i.p. with a dose of 1 ml/kg CCl_4_ (1:1 dilution in corn oil). The mice were sacrificed 24 h after CCl_4_ administration. Blood, spleen, and liver samples were collected for further analysis as following.

### Analysis of Serum Aspartate Aminotransferase and Alanine Aminotransferase Activity

The blood samples were collected and centrifuged to separate the serum. The activity of serum ALT and AST were determined by commercial reagent kit (AILEX, Shanghai, China) with a Hitachi 7600 automatic biochemical analyzer (Hitachi, Tokyo, Japan) according to the manufacturer’s protocol.

### Histopathological Analysis

The liver tissues from different groups of mice were fixed with 10% neutral buffered formalin and then embedded in paraffin. Paraffin-embedded tissues were cut into 5 µm thick sections and stained with hematoxylin and eosin for histopathological examination through a light microscope (Nikon, Tokyo, Japan). The percentage of necrotic area was determined by measuring the necrotic area relative to the entire liver area using Image J software (vision1.49, NIH, Bethesda, MD, USA) as described earlier (8).

### Flow Cytometry Analysis

Single cell suspensions prepared from spleen and liver were analyzed using flow cytometry (FACS) (28). The antibodies used for FACS staining including anti-mouse CD3-PE or CD3-FITC, CD4-PerCP/Cy5.5 or CD4-FITC, CD8-APC or CD8-FITC, CD69-FITC, CD62L-PerCP/Cy5.5, CD44-PE, CD19-APC, NK1.1-PE or NK1.1-PerCP/Cy5.5, Ly6C/Ly6G-Gr1, F4/80-PerCP/Cy5.5, IFNγ-PE, and TNFα-APC were purchased from Biolegend (San Diego, CA, USA). Anti-mouse CD16/32 FcR blocking antibody was also from Biolegend. For intracellular cytokine staining, cells were stimulated with 50 ng/ml phorbol 12-myristate 13-acetate (PMA, Beyotime, Haimen, China) and 500 ng/ml ionomycin (Beyotime) for 4 h in the presence of 1× brefeldin A (Biolegend). Cells were harvested and stained with surface antibodies, then were washed, fixed with 4% paraformaldehyde, permeabilized with 1% saponin and stained with intracellular cytokine antibodies. FACS was performed using a FACS Calibur flow cytometer (BD Biosciences, San Jose, CA, USA) and analyzed using Flowjo software (Tree Star, Ashland, OR, USA).

### Cytokine Analysis

Serum cytokine levels were analyzed using BD cytometric bead array (CBA) mouse inflammation kit (BD Pharmingen, San Diego, CA, USA) on a FACS Calibur Cytometer and analyzed using BD FCAP Array software (BD Bioscience). The cytokine kit covered mouse IL-6, IL-10, IL-12p70, TNF, IFNγ, and MCP-1.

### Reverse Transcription-Quantitative Real-Time PCR (RT-qPCR)

The total RNA was extracted from liver tissues with RNAiso plus reagent (TaKaRa, Dalian, China), and reverse-transcribed into cDNA with reverse transcriptase M-MLV reagent (TaKaRa, Dalian, China) according to manufacturer’s instructions. Quantitative real-time PCR (qPCR) was performed using SYBR Premix Ex Taq reagent (TaKaRa, Dalian, China) and carried out on a QuantStudio 3 real-time PCR system (Applied Biosystems, Foster City, CA, USA). The primer sequences used in the experiment were listed in supplementary Table S1 in Supplementary Material and the primers were synthesized by Genewiz Company (Suzhou, China). The β-actin gene was used as an internal control and the data were analyzed using 2^−ΔΔCt^ method.

### Immunofluorescence Staining

The sections of liver tissues were blocked with 3% BSA in PBS for 1 h at room temperature, and then incubated with primary antibodies overnight at 4°C. The primary antibodies were anti-Ly6C/Ly6G antibody (Bio X Cell, West Lebanon, NH), anti-F4/80 antibody (Abcam, Cambridge, MA, USA) and anti-fibronectin antibody (Abcam). After washing in PBS for three times, the sections were incubated with Alexa Fluor 555 anti-rat IgG antibody or Alexa Fluor 488 anti-rabbit IgG antibody (Abcam) at room temperature for 1 h in the dark. After counterstaining with DAPI (Beyotime), the sections were observed and photographed with a Leica TCS SP8 Confocal Microscope (Buffalo Grove, IL, USA).

### TUNEL Staining

To evaluate apoptosis of hepatocytes in liver tissues, paraffin-embedded sections were stained by using a One Step TUNEL Apoptosis Assay Kit (Beyotime) according to the manufacturer’s instructions. Sections were counterstained with DAPI and observed with a Leica TCS SP8 confocal microscope.

### Western Blot Analysis

Total proteins were extracted from liver tissues with the strong RIPA lysate kit (Beyotime), and the protein concentration was determined by BCA protein method. Proteins were denatured, separated by SDS-PAGE gel, and transferred to polyvinylidene difluoride membranes. The membranes were blocked with 5% non-fat powdered milk or 3% BSA in TBST for 2 h at room temperature and incubated with primary antibodies overnight at 4°C. The primary antibodies used in this study were against ERK, p-ERK, p38, p-p38, JNK, p-JNK, AKT, p-AKT, NF-κB p65, Bax, Bcl-2 (all from Cell Signaling Technology, Danvers, MA, USA), MXRA7 (Sigma, St. Louis, MO, USA), GAPDH (SUNGENE, Tianjin, China), and β-actin (ImmunoWay, Plano, TX, USA). Following washes with TBST three times, the membranes were incubated with HRP-conjugated goat anti-rabbit IgG or goat anti-mouse IgG antibody (ImmunoWay) for 1.5 h at room temperature. After washing off the unbound antibody three times with TBST, the protein bands were detected by an enhanced chemiluminescence kit (Beyotime) using a CLiNX Science Instrument (Shanghai, China). The intensity of the target protein bands was calculated using Image J software.

### Statistical Analysis

All data, when appropriate, are presented as mean ± SD with sample size (as indicated as *n*=) and repeated times of experiments detailed in figure legends. Statistical analysis was performed using the Graphpad prism 5 software (Graphpad, San Diego, CA, USA). One-way of ANOVA analysis and Student’s *t*-test (unpaired, two-tailed) were used for statistical analysis. *p* < 0.05 was considered to be statistically significant (*), <0.01 or 0.001 was shown as ** or ***, respectively.

## Results

### MXRA7 Deficiency Alleviates While MXRA7 Overexpression Aggravates CCl_4_-Induced ALI in Mice

**Figure 1:**
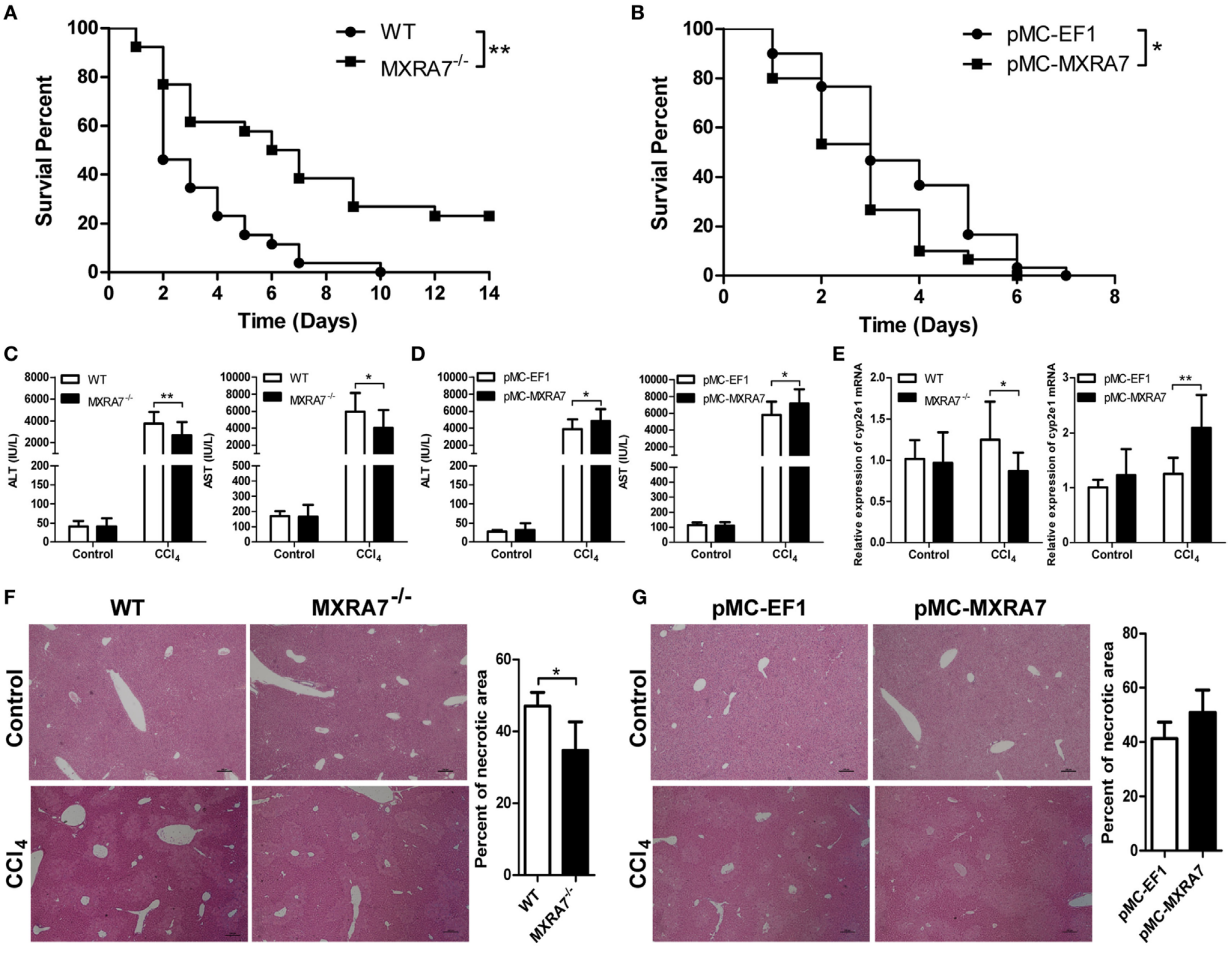
Matrix remodeling associated 7 (MXRA7) deficiency alleviates, while MXRA7 overexpression aggravates CCl_4_-induced acute liver injury. **(A)** Wild-type (WT) and MXRA7^−/−^ mice were administered intraperitoneally with 6 ml/kg CCl_4_ (1:1 corn oil), and survival of animals were monitored daily for 14 days after *n* = 26 in each group. The data was expressed as the Kaplan–Meier survival curves and analyzed by the log-rank test. The data shown are the summary of three experiments. **(B)** C57BL/6 mice were injected with mock pMC-EF1 (control) or pMC-MXRA7 plasmids by hydrodynamic gene transfer method. Three days later, the animals were challenged with CCl_4_ as above. Survivals were monitored once a day for 7 days after administration. *n* = 30 each group. The data shown are the summary of four experiments. **(C–G)** WT and MXRA7^−/−^ mice, or pMC-EF1 and pMC-MXRA7-pulsed mice, were treated with 1 ml/kg CCl_4_ injection (1:1 corn oil) or control oil. Twenty four hours later, the animals (*n* = 6 for control group, *n* = 18–23) were sacrificed and serum were harvested measurement of ALT and AST **(C,D)**, while livers (*n* = 3 for control group, *n* = 9 for CCl_4_) were harvested and subjected to measurement of CYP2E1 mRNAs using reverse transcription-quantitative real-time PCR **(E)** and structure evaluation after histological hematoxylin and eosin staining **(F,G)**. For histological evaluation, necrotic areas were determined by microscopy. The percentage of necrotic area was determined by dividing the sum area of necrosis by the sum of the total liver area of four fields. One representative section is shown for each group (original magnification: 40×). The data shown are the summary of three experiments. *p < 0.05, **p < 0.01.

To evaluate the hypothetical role of MXRA7 in murine ALI model, WT and MXRA7^−/−^ mice, as well as pMC-MXRA7 mediated MXRA7-overexpressing mice, were compared. The overexpression of MXRA7 in the HGT-pulsed liver was confirmed at both mRNA and protein levels (Figure S2 in Supplementary Material). Compared with controls in WT mice, deficiency of MXRA7 prolonged the survival of mice after CCl_4_ treatment (Figure 1A), while the overexpression of MXRA7 in liver slightly but statistically significantly increased the mortality of mice (Figure 1B). To further confirm the effect of MXRA7 on ALI, the levels of ALT and AST in serum were measured, and histopathological phenotypes in liver were examined. While both transferases were significantly increased in serum in both WT and MXRA7^−/−^ mice upon CCl_4_ treatment, the deficiency of MXRA7 markedly impaired the increase of serum ALT and AST (Figure 1C), while the overexpression of MXRA7 increased the amounts of serum ALT and AST (Figure 1D). As a marker enzyme in the process of CCl_4_-induced liver injury, expression of CYP2E1 in livers of these mice was compared. It was found that MXRA7 deficiency decreased while MXRA7 overexpression increased the expression of CYP2E1, respectively (Figure 1E). When the livers were subjected to histological analysis, the necrotic areas around the central vein and centrilobular regions in CCl_4_-treated MXRA7^−/−^ mice were significantly decreased when compared with that in WT mice (Figure 1F), and MXRA7 overexpression manifested an opposite effect to that of MXRA7 deficiency (Figure 1G). These findings indicated that MXRA7 overexpression promoted ALI development in CCl_4_-challenged mice, while MXRA7 deficiency protected mice from such injury.

### MXRA7 Deficiency Decreases Neutrophils and Macrophages Cells Infiltration in Liver

**Figure 2:**
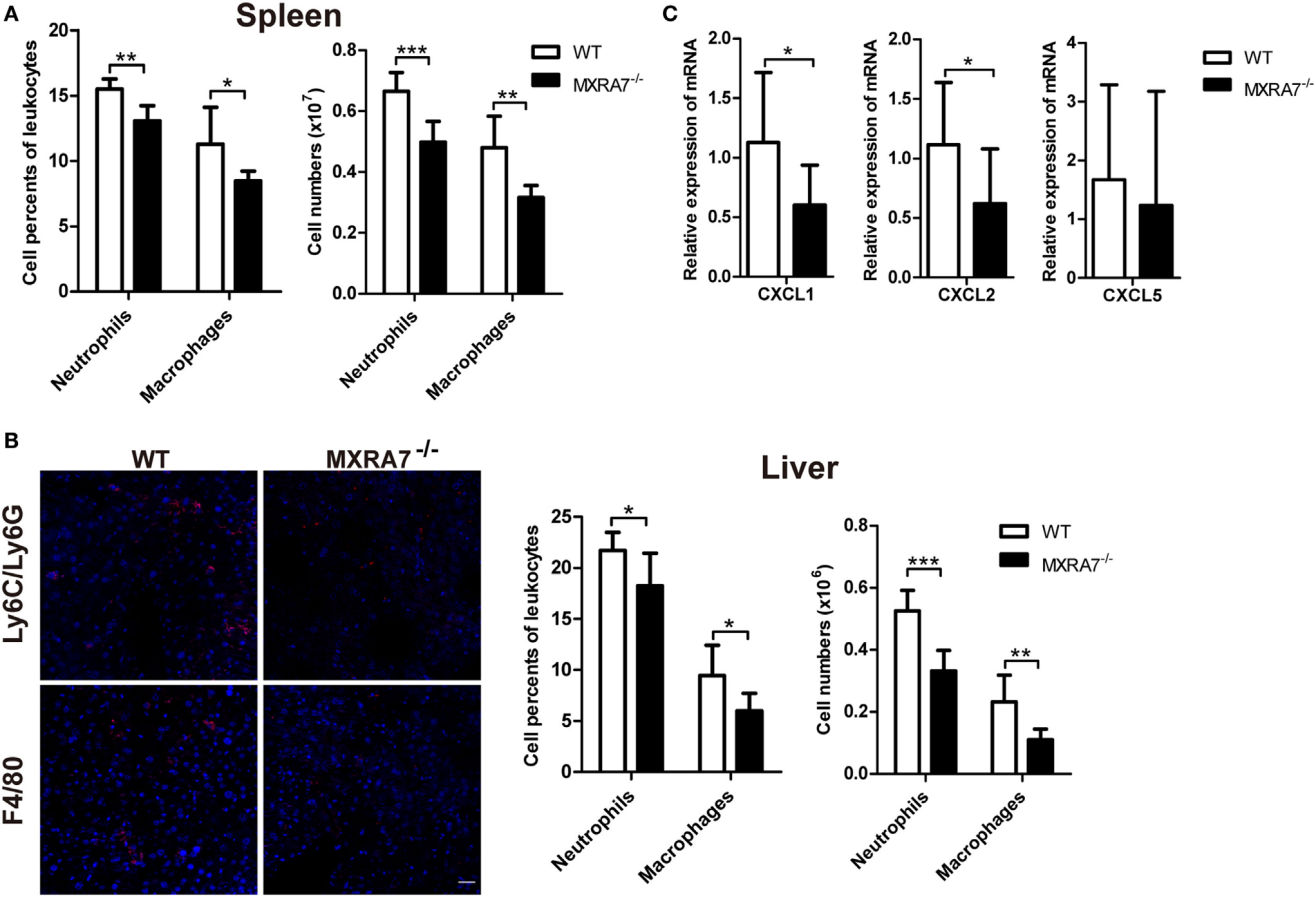
Matrix remodeling associated 7 (MXRA7) deficiency decreases neutrophils and macrophages infiltration in liver. **(A,B)** Splenocytes and intrahepatic leukocytes were isolated from 1 ml/kg CCl_4_-challenged wild-type (WT) and MXRA7^−/−^ mice (*n* = 7 each group) 24 h after CCl_4_ injection and used for FACS analysis. **(B)** Immunofluorescence staining of Ly6C/Ly6G (in red) and F4/80 (in red) in liver sections from CCl_4_-treated WT and MXRA7^−/−^ mice. Scale bar = 25 µm. Ly6C/Ly6G^+^ neutrophils and F4/80^+^ macrophages in spleen **(A)** and in liver **(B)** were measured. Data shown are the representative of three independent experiments. **(C)** Livers were harvested from mice which were sacrificed 24 h after 1 ml/kg CCl_4_ injection. Expression of CXCL1, CXCL2, and CXCL5 mRNAs in livers was detected by reverse transcription-quantitative real-time PCR. The data shown are the summary of three experiments. *p < 0.05, **p < 0.01.

The inflammatory cells play important roles in the development of liver injury or related liver diseases, among which neutrophils and macrophages are two classes of well-known players (13, 29, 30). In order to investigate the effect of MXRA7 on the inflammatory cells during liver injury, we examined the inflammatory cells in spleen and liver by flow cytometry or immunofluorescence staining. The deficiency of MXRA7 significantly decreased the percentage and cell number of Ly6C/Ly6G^+^ neutrophils and F4/80^+^ macrophages in both spleens and livers (Figures 2A,B). Furthermore, the expressions of CXCL1 and CXCL2, two well-documented neutrophils chemoattractants, were significantly decreased in MXRA7^−/−^ mice (Figure 2C). However, MXRA7 overexpression had no effect on the recruitment of neutrophils and the expression of associated chemokines (Figure S3 in Supplementary Material). These results indicated that deficiency of MXRA7 reduced the expression of CXCL1 and CXCL2, presumably suppressed the recruitment of neutrophils.

### MXRA7 Deficiency Decreases the Numbers of CD8^+^ T Cells and Secretion of IFNγ and TNFα

**Figure 3:**
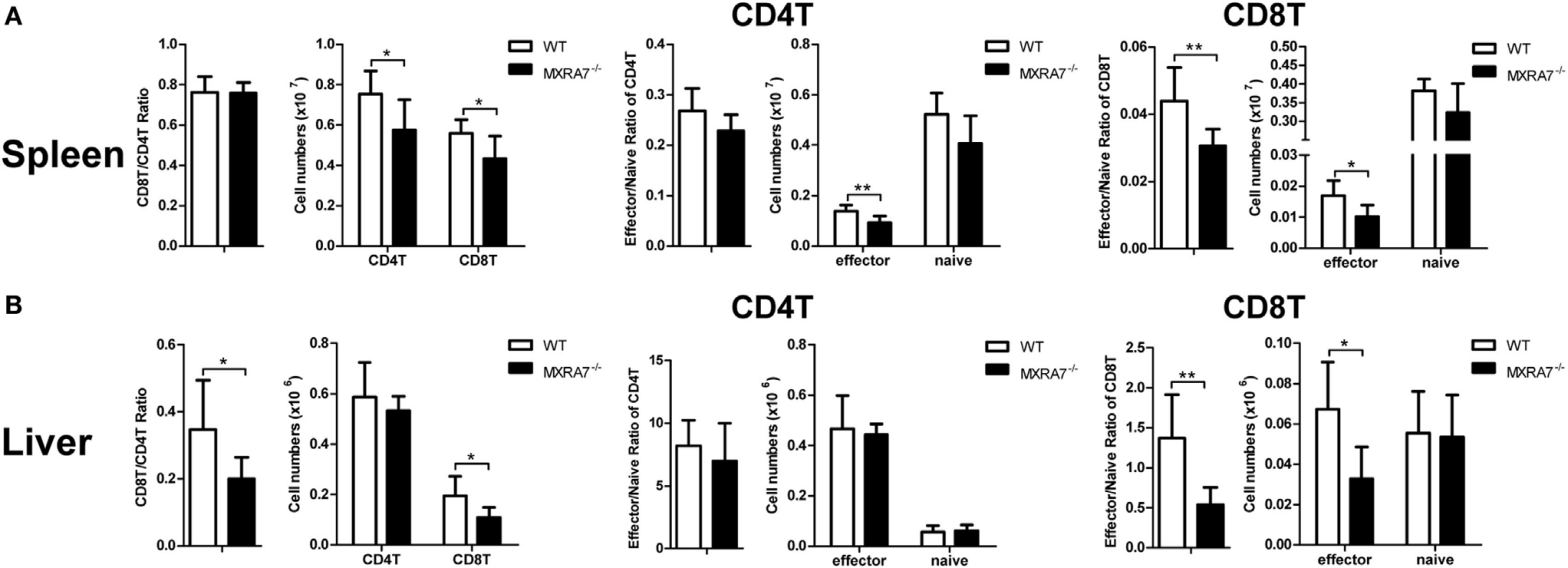
Matrix remodeling associated 7 (MXRA7) deficiency decreases the numbers of CD8^+^ T cells. Mice from wild-type (WT) and MXRA7^−/−^ groups (*n* = 7 each group) were sacrificed 24 h after 1 ml/kg CCl_4_ injection. Splenocytes and intrahepatic leukocytes were isolated for FACS analysis. **(A)** Flow cytometric analysis showing slight but statistically significant decrease of CD8^+^ T cells and effector cells (CD62L^-^CD44^+^) in CD4^+^ T, CD8^+^ T cells in spleen. **(B)** Flow cytometric analysis of CD4^+^ T and CD8^+^ T cells, and effector cells (CD62L^−^CD44^+^) in liver. Data shown are the representative of three independent experiments. *p < 0.05, **p < 0.01.

**Figure 4:**
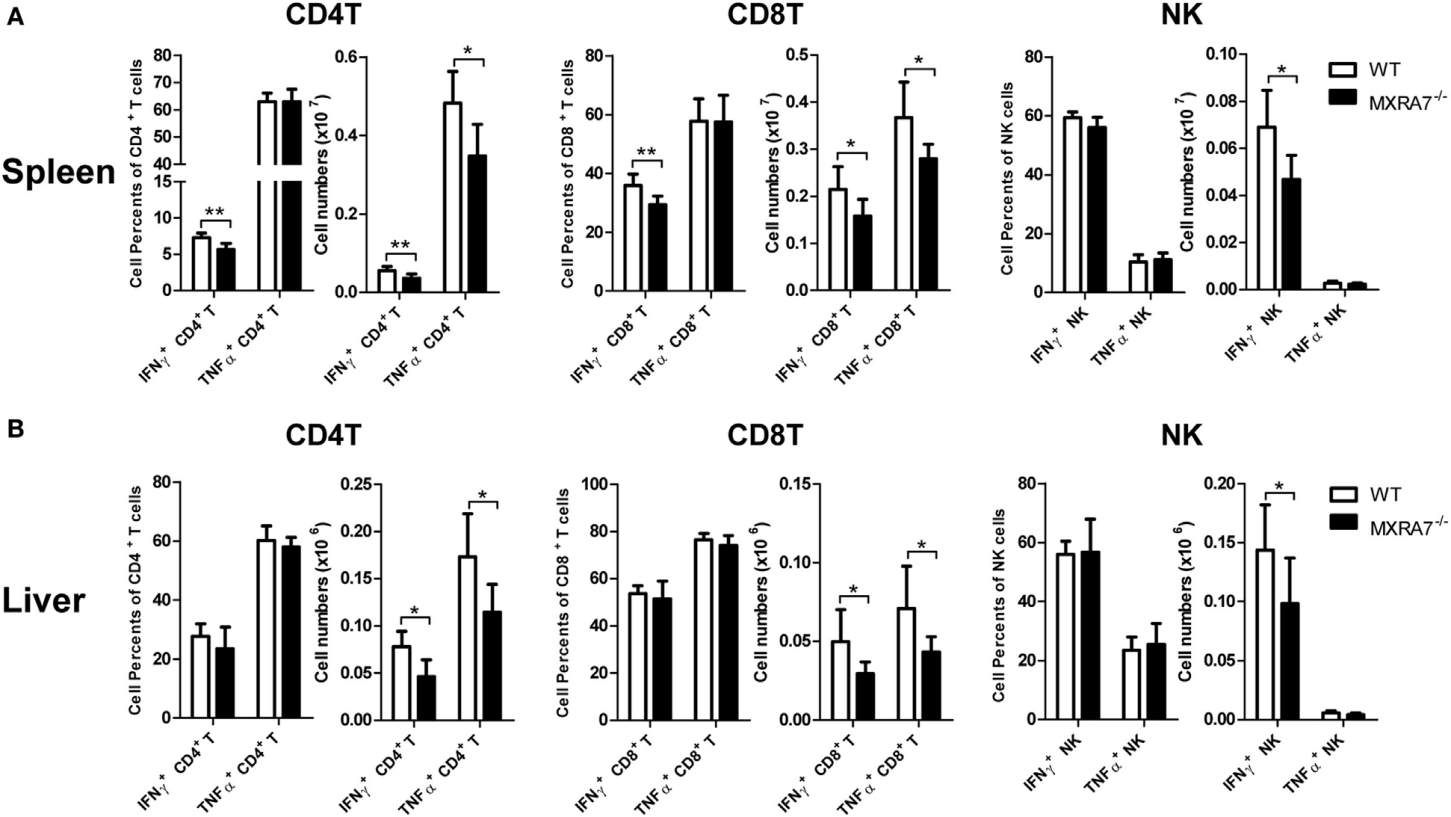
Matrix remodeling associated 7 (MXRA7) deficiency inhibits IFNγ and TNFα production in T cells. Twenty four hours after 1 ml/kg CCl_4_ injection, mice from different groups (*n* = 7 each group) were sacrificed. Splenocytes **(A)** and intrahepatic leukocytes **(B)** were isolated, stimulated, and stained for intracellular cytokines in combination with cell surface staining. The percentages of IFNγ^+^ in CD4^+^ T and CD8^+^ T cells were decreased in MXRA7^−/−^ spleens. The numbers of IFNγ^+^ and TNFα^+^ in CD4^+^ T and CD8^+^ T cells were decreased in both spleens and livers of MXRA7^−/−^ mice. Data shown are the representative of three independent experiments. *p < 0.05, **p < 0.01.

T cells had been suggested as both targets and effector cells in CCl_4_-induced injury (31). To check if MXRA7 participated in liver injury processes *via* the immune compartment in above liver injury model, T cells in liver as well as spleen were analyzed by flow cytometry. Compared with WT group, MXRA7 deficiency reduced the numbers of CD4^+^ T cells and CD8^+^ T cells, and the effector cells (CD62L^−^CD44^+^) in spleens (Figure 3A). MXRA7 deficiency also reduced CD8^+^/CD4^+^ ratio and the number of CD8^+^ T cells, while decreased CD8^+^ T effector cells in livers (Figure 3B). Since T or NK cells-derived cytokines were responsible for induction of inflammation that aggravates liver injury (32, 33), we examined IFNγ and TNFα secretion by T and NK cells. While the percentages and cell numbers of CD4^+^ T and CD8^+^ T producing IFNγ were significantly decreased in spleens of MXRA7^−/−^ mice (Figure 4A), the cell numbers of CD4^+^ T and CD8^+^ T producing IFNγ and TNFα were decreased in livers of MXRA7^−/−^ mice as well (Figure 4B). The numbers of NK cells producing IFNγ decreased in both spleens and livers of MXRA7^−/−^ mice (Figures 4A,B), while the T cells and production of IFNγ and TNFα showed no difference between MXRA7 overexpression and control group (Figures S4 and S5 in Supplementary Material). These data suggested that MXRA7 deficiency might alleviate liver injury by reducing CD8^+^ T cells and suppressing the production of IFNγ and TNFα from T cells.

### MXRA7 Deficiency Reduces the Expression of Pro-Inflammatory Cytokines in ALI Model

**Figure 5:**
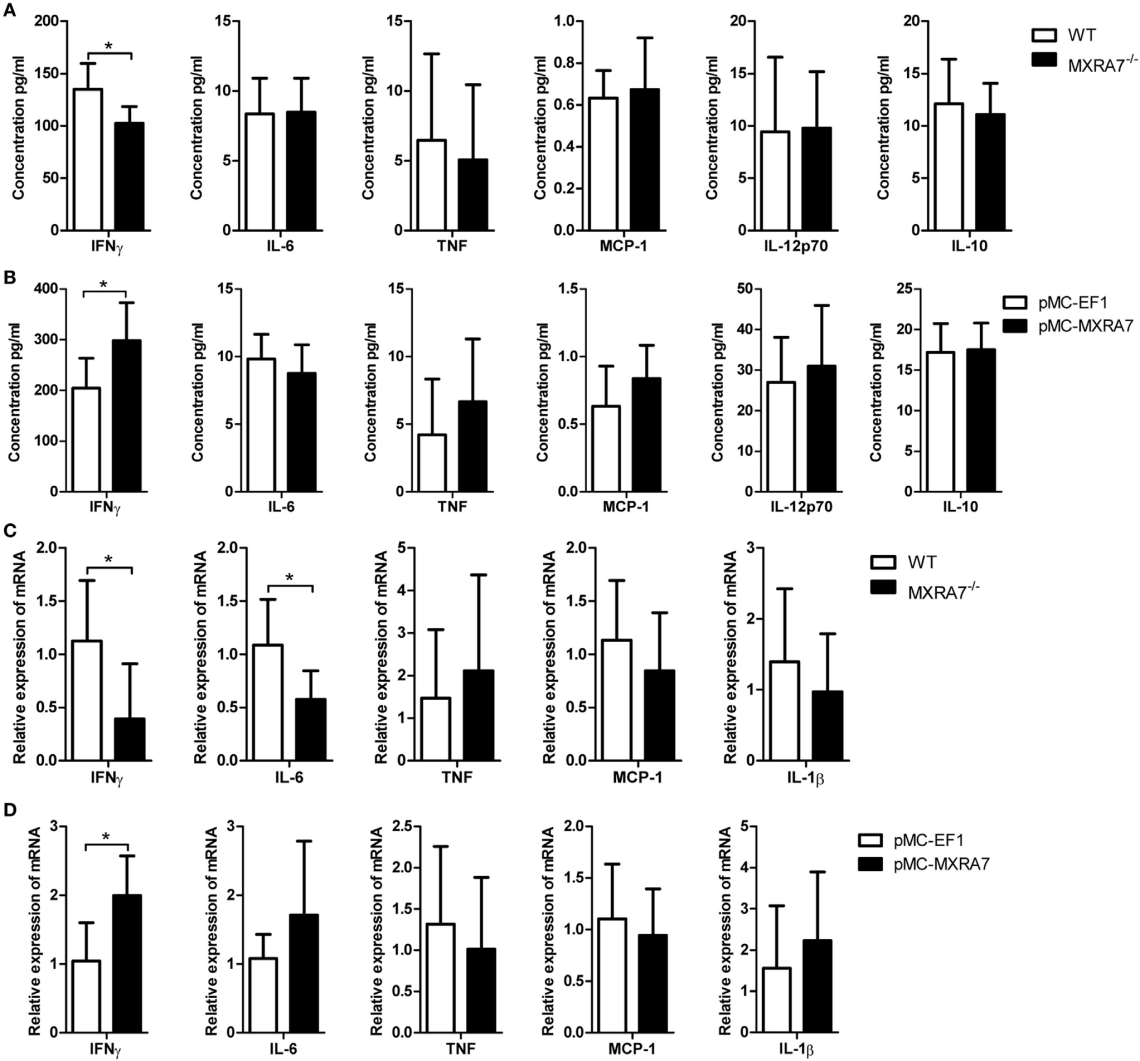
Matrix remodeling associated 7 (MXRA7) deficiency inhibits and MXRA7 overexpression promotes the expression of pro-inflammatory cytokines. **(A,B)** Serum was collected from different groups of mice 24 h after 1 ml/kg CCl_4_ injection. Serum IFNγ, IL-6, TNF, MCP-1, IL-12p70, and IL-10 were determined using cytometric bead array mouse inflammation kit. **(C,D)** Livers were isolated from different groups of mice 24 h after 1 ml/kg CCl_4_ injection. Expression of IFNγ, IL-6, TNF, MCP-1, and IL-1β mRNAs in livers was detected by reverse transcription-quantitative real-time PCR. Data are presented as the summary of three experiments. *p < 0.05.

The inflammatory cytokines of serum IFNγ, IL-6, TNF, MCP-1, IL-12p70, and IL-10 were determined by CBA mouse inflammation kit. The deficiency of MXRA7 decreased the level of serum IFNγ (Figure 5A), and the overexpression of MXRA7 increased IFNγ level (Figure 5B). Moreover, we also measured the expression of pro-inflammatory cytokines in livers. The mRNA expressions of IFNγ and IL-6 were significantly decreased in MXRA7^−/−^ group when compared to WT group (Figure 5C), while MXRA7 overexpression elevated the mRNA expression of IFNγ (Figure 5D). None of other tested cytokines or their genes manifested significant change. These results suggested that IFNγ was the main pro-inflammatory cytokines mediating the differential responses to CCl_4_-induced injury in mice under different MXRA7 context.

### MXRA7 Deficiency Inhibits While MXRA7 Overexpression Promotes the Expression of Fibronectin and TIMP1

**Figure 6:**
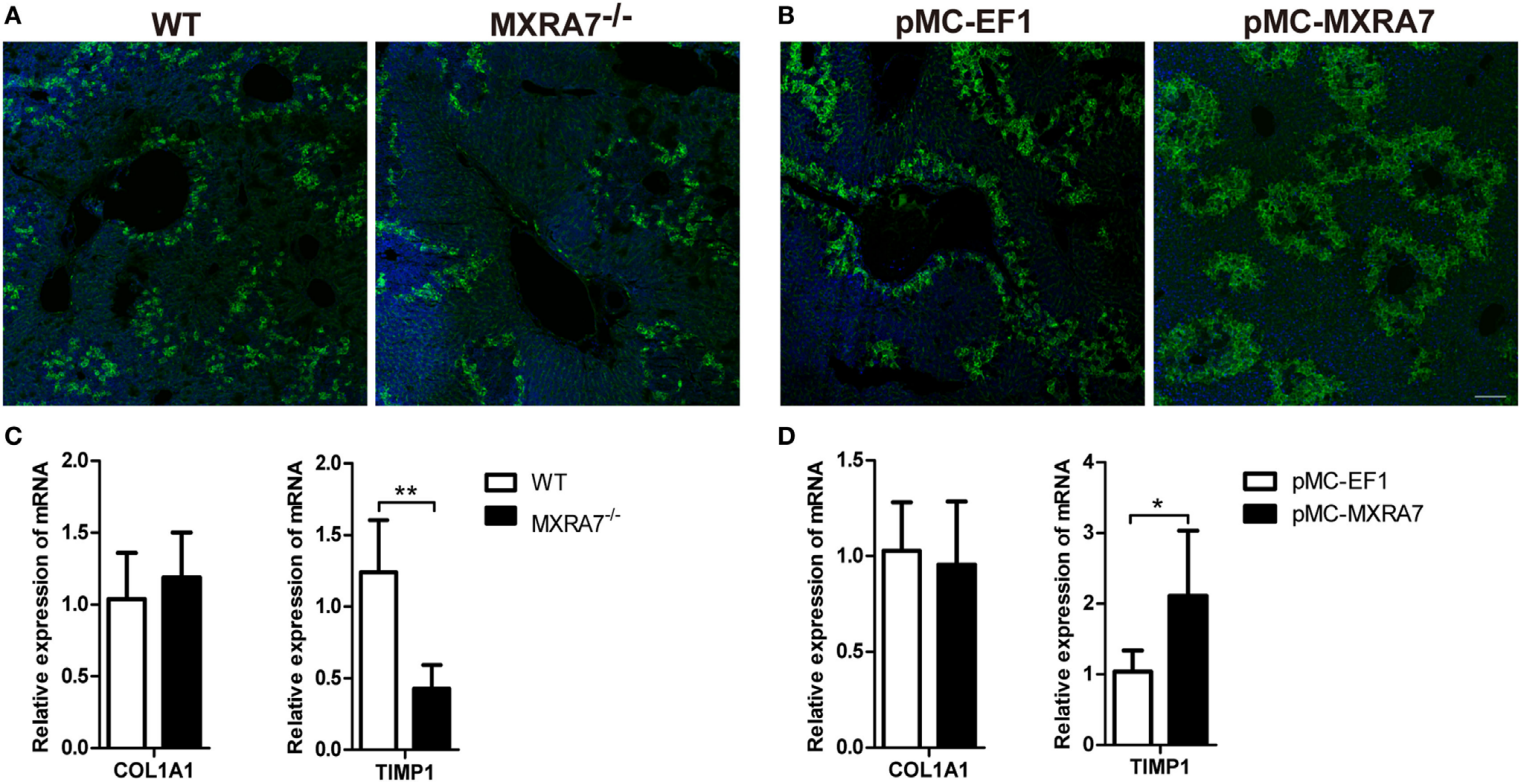
Matrix remodeling associated 7(MXRA7) deficiency inhibits and MXRA7 overexpression promotes the expression of fibronectin and TIMP1. Livers were isolated from different groups of mice 24 h after 1 ml/kg CCl_4_ injection. **(A,B)** Expression of fibronectin (in green) in livers was detected using immunofluorescence analysis. Representative images from different groups are shown. Scale bar = 100 μm. **(C,D)** Expression of COL1A1 and TIMP1 mRNAs in livers was detected by reverse transcription-quantitative real-time PCR. Data are presented as the summary of two experiments. *p < 0.05, **p < 0.01.

Liver injury induces inflammation and expression of extracellular matrix proteins, such as fibronectin and collagen I (COL1A1) (34, 35). Immunofluorescence staining showed that MXRA7 deficiency decreased the expression of fibronectin in liver compared to WT mice (Figure 6A), while MXRA7 overexpression increased fibronectin expression (Figure 6B). However, MXRA7 had no effect on the expression of COL1A1 (Figures 6C,D). Tissue inhibitor of metalloproteinase 1 was also tested since it is a well-documented modulator of matrix remodeling (36). MXRA7^−/−^ mice showed lower expression of TIMP1 mRNA than WT mice, and MXRA7 overexpression increased the mRNA level of TIMP1 (Figures 6C,D). These data suggest that MXRA7 plays a role in the reconstruction of extracellular matrix.

### MXRA7 Deficiency Suppresses the Cell Apoptosis Pathway and Inflammatory Pathway

**Figure 7:**
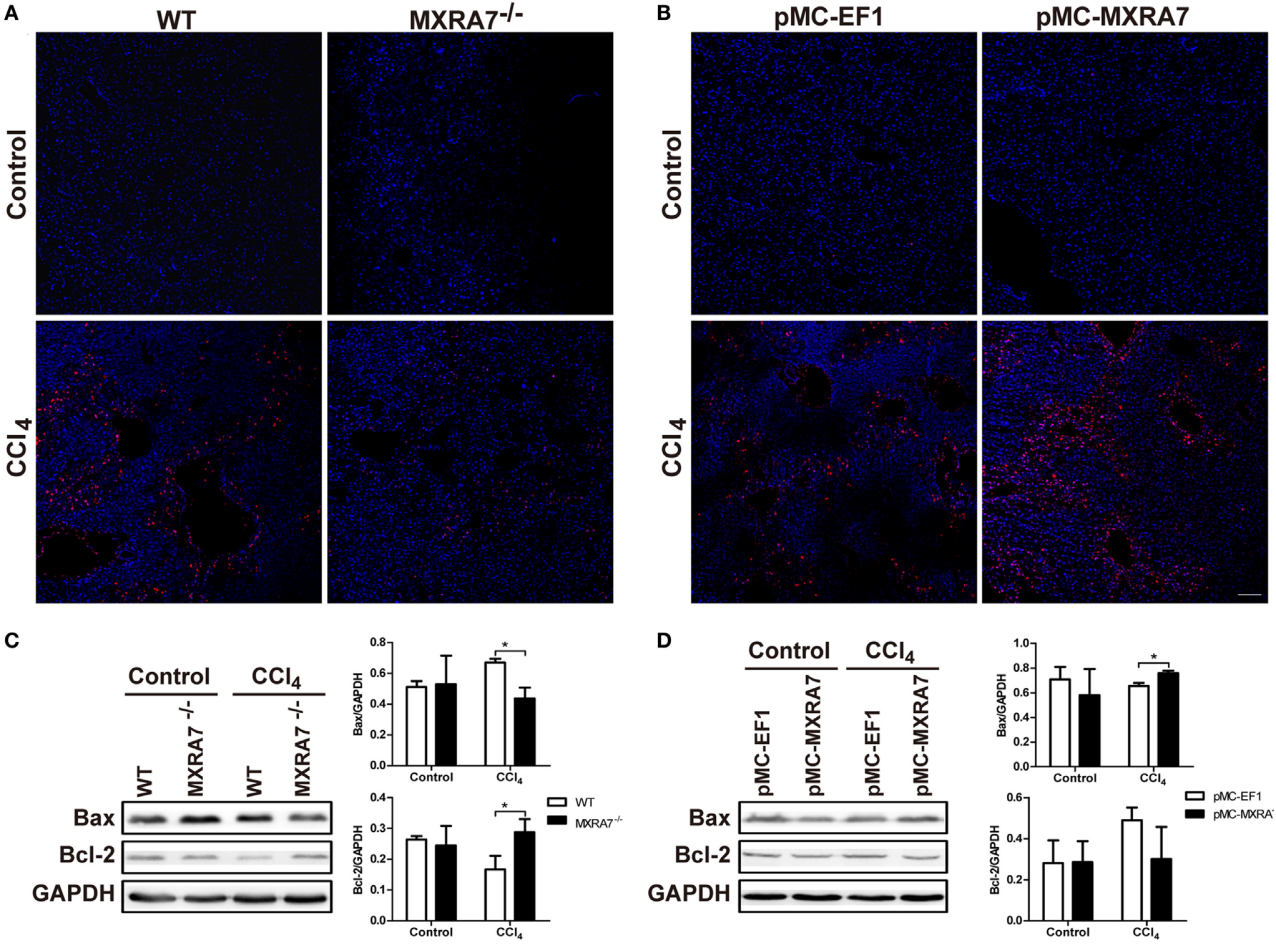

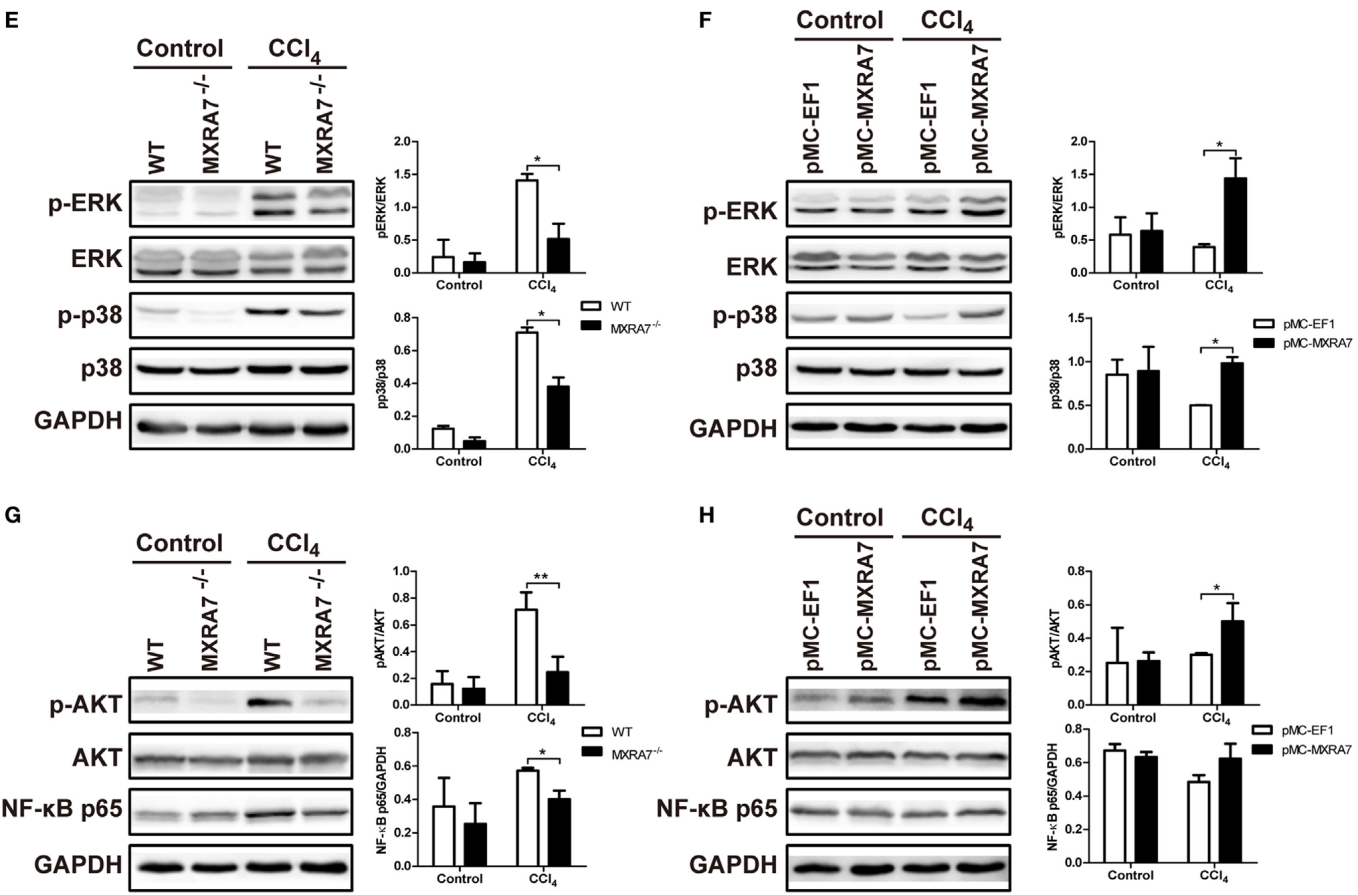
Matrix remodeling associated 7 (MXRA7) deficiency suppresses the cell apoptosis pathway and inflammatory pathway in livers. Livers were harvested from different groups of mice 24 h after 1 ml/kg CCl_4_ injection. **(A,B)** Hepatocyte apoptosis in liver was analyzed using TUNEL staining (in red). Representative images from different groups are shown. Scale bar = 100 μm. And proteins were extracted from livers for western blot analysis of Bax and Bcl-2 **(C,D)** p-ERK, ERK, p-p38, p38 **(E,F)**, p-AKT, AKT, and NF-κB p65 **(G,H)**. Representative images shown are the representative of three independent experiments. *p < 0.05, **p < 0.01.

To clarify whether MXRA7 could affect the apoptosis of hepatocytes, the TUNEL assay was performed. MXRA7 deficiency inhibited cell apoptosis and MXRA7 overexpression promoted cell apoptosis upon CCl_4_ treatment (Figures 7A,B). The expressions of Bcl-2 and Bax in livers were also compared among different groups. After CCl_4_ treatment, the protein expressions of the proapoptotic protein Bax were decreased in livers from MXRA7^−/−^ mice, while the levels of the antiapoptotic factor Bcl-2 was significantly increased (Figure 7C). On the contrary, MXRA7 overexpression increased the expression of Bax as compared to control group (Figure 7D).

To understand the underlying molecular mechanisms of the effects of MXRA7 on CCl_4_-induced liver injury, we examined the MAPK and AKT/NF-κB signaling pathways, which are known to regulate cell apoptosis and inflammation. Western blot analysis showed that MXRA7 deficiency reduced the phosphorylated protein levels of ERK and p38 when compared to WT mice (Figure 7E), while MXRA7 overexpression increased the levels of p-ERK and p-p38 after CCl_4_ treatment (Figure 7F). However, MXRA7 deficiency or overexpression did not show significant effect on phosphorylation of JNK (Figure S6 in Supplementary Material). MXRA7 deficiency also decreased the expression of p-AKT and NF-κB p65 (Figure 7G), while MXRA7 overexpression increased the expression of p-AKT after CCl_4_ treatment (Figure 7H). Thus, the protective roles of MXRA7 deficiency against CCl_4_-induced hepatocyte apoptosis and inflammation might be associated with suppressing MAPK and AKT/NF-κB pathways.

## Discussion

Since named in 2002 (22), MXRA7 had been just mentioned in some studies without any purpose investigation into its biological functions. Previous studies noted that MXRA7 was overexpressed in childhood acute lymphoblastic leukemia and in ovarian endometriomas (37, 38). In an effort to characterize the functions of MXRA7, our lab had found that MXRA7 was involved in the pathological process of ocular inflammatory models (21) and MXRA7 might play a role in tissue injury, wound healing, and cancer (manuscripts under review).

In the current study, the potential role of MXRA7 in liver disease was investigated in mice by utilizing the CCl_4_-induced liver injury model. This model has been widely used to study both acute injury and following chronic fibrosis (39–41). We found that MXRA7 overexpression aggravated liver injury, and MXRA7 deficiency protected the liver against CCl_4_-induced liver injury (Figure 1). Serum AST and ALT levels, which were often used as indicators of hepatic damage and functional integrity of liver (42), manifested changes in line with the animal survival or histological changes. CCl_4_ could induce liver damage through CYP2E1 (43), though the necrotic area would possibly not produce CYP2E1 anymore, MXRA7 might affect liver injury partly by regulating the level of CYP2E1. In summary, all these results indicated a hepatoprotective effect of MXRA7 deficiency on CCl_4_-induced ALI. In another word, MXRA7 might be a positive modulator of CCl_4_-induced ALI.

Though the main functions of the liver are focused on metabolism, it is also an immunologic organ since the immune cells resided in the liver are involved in the development of inflammation and fibrosis when responding to various insults (44). In CCl_4_-induced liver inflammation and injury model, lymphocytes, neutrophils, and macrophages in liver tissues play important roles through secreting cytokines and chemokines (13, 45–47). Previous study reported that CD8^+^ T cells could induce more liver injury and fibrosis in mice treated with CCl_4_, and CD4^+^/CD8^+^ ratio reduction also involved in induction of liver fibrosis in human (40, 48). The data presented here demonstrated that MXRA7 deficiency not only impaired infiltration of neutrophils and marcrophages in liver induced by CCl_4_ (Figure 2) but also decreased the number of CD8^+^ T cells and CD8^+^/CD4^+^ ratio in liver of MXRA7^−/−^ mice compared to WT mice (Figure 3). In line with this observation, MXRA7 deficiency suppressed the production of IFNγ and TNFα in T cells (Figure 4) and suppressed the expression (at mRNA level) of IFNγ and IL-6 in liver, hence decreased the protein level of IFNγ in serum. Meanwhile, MXRA7 overexpression increased the IFNγ mRNA in liver and protein in serum. Collectively, these data showed that when MXRA7 was deficient in animals, CCl_4_-induced inflammatory or immune cells activation were decreased, leading to an alleviated liver injury than in WT host receiving same challenge.

Lastly, cell apoptosis and inflammatory signaling pathways associated with CCl_4_-induced injury (49) were measured. MXRA7 deficiency suppressed cell apoptosis, decreased Bax expression, and increased Bcl-2 expression, while MXRA7 overexpression promoted cell apoptosis and upregulated the expression of Bax (Figure 7). When the hepatic apoptosis pathways, e.g., MAPK signaling pathway, were compared in context of ALI models, the phosphorylation levels of ERK and p38 regulating cell apoptosis were downregulated in MXRA7^−/−^ mice and upregulated in MXRA7 overexpression mice. AKT/NF-κB pathway has dual function in pro-inflammatory and cell survival, the unbalance of NF-κB activation may cause increased inflammation or insufficient protection from cell apoptosis (50, 51). However, the effect of MXRA7 deficiency or overexpression on the AKT phosphorylation or p65 expression was a little bit complicated in CCl_4_-induced ALI. The overall impression was that MXRA7 deficiency depressed AKT phosphorylation and NF-κB p65 expression while MXRA7 overexpression increased AKT phosphorylation (Figure 7). These results suggested that in the studied ALI model, MXRA7 not only mediated liver injury *via* the inflammation or immune compartment but also *via* acting in/on hepatocytes directly or indirectly.

In summary, this study represented the first effort to explore the possibility that the seldom-addressed gene MXRA7 was involved in ALI. Using genetically MXRA7-deficient mice and assisted with HGT-mediated liver-specific overexpression of MXRA7, we were able to confirm that MXRA7 played a positive role in initiation of CCl_4_-induced ALI. It will be of interest to investigate whether such a hypothesis for MXRA7 function was applicable with other acute liver injuries, or even with chronic liver injuries. For example, repeated injection of CCl_4_ in mice leads to chronic liver fibrosis, a process that is more closely related with matrix remodeling. This said, though the results that MXRA7 deficiency decreased and MXRA7 overexpression increased the expression of fibronectin and TIMP1 in murine livers (Figure 6) were still preliminary, they strongly implied that MXRA7 might also be involved in such chronic liver injuries as those induced by repeated CCl_4_ challenge, heavy alcohol consumption, drug intoxication, or consistent virus infections. Should all these hypotheses be confirmed by future studies, MXRA7 might serve a promising new therapeutic target for preventing or treating those liver injuries. However, in spite of the current data demonstrating the potential significance of MXRA7 in liver injury, much more investigations are guaranteed to help fully understand the role of MXRA7 in ALI, ALI-based diseases, or in overall physiology or pathology of the liver.

## Ethics Statement

All animal experiments were carried out in accordance with the Guidelines on the Humane Treatment of Laboratory Animals (Ministry of Science and Technology of China, 2006) and approved by Ethical Committee of The First Affiliated Hospital of Soochow University. All measures were taken to ensure the humane treatment of research animals.

## Author Contributions

YW and DL designed the study and wrote the manuscript. DL, ZS, ZJ, LL, YL, BH, BW, and YS performed the experiments. DL, ZS, and ZJ analyzed the data.

## Conflict of Interest Statement

The authors declare that the research was conducted in the absence of any commercial or financial relationships that could be construed as a potential conflict of interest.
