# Supplementary material for: Matrix Remodeling Associated 7 Deficiency Alleviates Carbon Tetrachloride-Induced Acute Liver Injury in Mice
Source: Front Immunol. 2018 Apr 18;9:773. doi: 10.3389/fimmu.2018.00773 (PMC5915751; doi:10.3389/fimmu.2018.00773)
Supplement: Supplementary file 1 [file data_sheet_1.docx]

Supplementary Material

MXRA7 deficiency alleviates carbon tetrachloride-induced acute liver injury in mice

Dandan Lin, Zhenjiang Sun, Ziqi Jin, Lei Lei, Yonghao Liu, Bo Hu, Benfang Wang, Ying Shen, Yiqiang Wang^*^

*** Correspondence:** Yiqiang Wang, 708 Renmin Road, Suzhou, 215007, China. Tel: +86-512-67780780; Email: yiqiangwang@suda.edu.cn

# Supplementary Tables

**Table S1. Primer sequences used for RT-qPCR.**

| Gene | Primer sequence |
| --- | --- |
| MXRA7 | F: 5′- CCACCCACTGAAGAACCTGA-3′ |
|  | R: 5′-TCGCCAAAGGTGTCCTTGTT-3′ |
| CYP2E1 | F: 5′-AGTACAAGAACAAGGGGATT-3′ |
|  | R: 5′-AGAAAGGTAGGGTCAAAAGG-3′ |
| CXCL1 | F: 5′- ACTCAAGAATGGTCGCGAGG-3′ |
|  | R: 5′- GTGCCATCAGAGCAGTCTGT-3′ |
| CXCL2 | F: 5′- TTGCCTTGACCCTGAAGCCCCC-3′ |
|  | R: 5′-GGCACATCAGGTACGATCCAGGC-3′ |
| CXCL5 | F: 5′- CGGTTCCATCTCGCCATTCA-3′ |
|  | R: 5′- GCGGCTATGACTGAGGAAGG-3′ |
| IFNγ | F: 5′- CGGCACAGTCATTGAAAGCCTA-3′ |
|  | R: 5′- GTTGCTGATGGCCTGATTGTC-3′ |
| IL-6 | F: 5′-ACCAGAGGAAATTTTCAATAGGC-3′ |
|  | R: 5′-TGATGCACTTGCAGAAAACA-3′ |
| TNFα | F: 5′- CCCTCCTGGCCAACGGCATG-3′ |
|  | R: 5′- TCGGGGCAGCCTTGTCCCTT-3′ |
| MCP-1 | F: 5′-GGCTGGAGAGCTACAAGAGG-3′ |
|  | R: 5′- GGTCAGCACAGACCTCTCTC-3′ |
| IL-1β | F: 5′-GCCTCGTGCTGTCGGACCCATAT-3′ |
|  | R: 5′-TCCTTTGAGGCCCAAGGCCACA-3′ |
| COL1A1 | F: 5’-CTGACTGGAAGAGCGGAGAGTAC-3’ |
|  | R: 5’-GGTCAGCTGGATAGCGACATC-3’ |
| TIMP1 | F: 5’-GGACCTGGTCATAAGGGCTA-3’ |
|  | R: 5’-GGCATATCCACAGAGGCTTT-3’ |
| β-actin | F: 5′- GCTCCTAGCACCATGAAGAT-3′ |
|  | R: 5′- GTGTAAAACGCAGCTCAGTA-3′ |

# Supplementary Figures

**
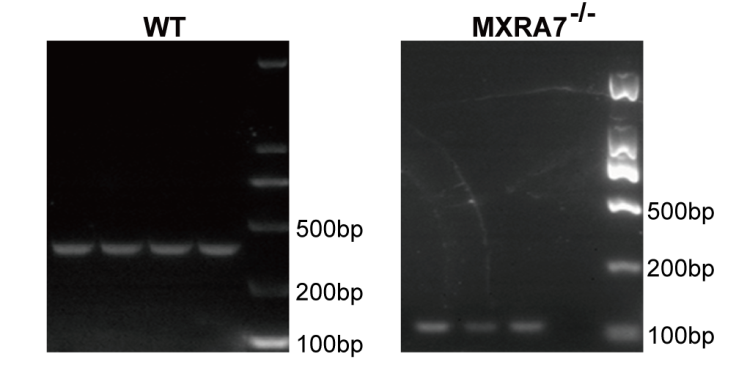
**

**Figure S1. Genotyping of wild type or MXRA7^-/-^ mice by PCR analysis of genomic DNA.** Primers used for genotyping were as follows: MXRA7, 5’-ACATCTCTCTAGCCCCAGGT-3’ (forward), and 5’-CACACCCTCTAGTCCCACAT-3’ (reverse); and lac Z, 5’-ATCACGACGCGCTGTATC-3’ (forward) and 5’-ACATCGGGCAAATAATATCG-3’ (reverse). The PCR product of WT mice was 403 bp (for MXRA7), and the PCR product of MXRA7^-/-^ mice was 108 bp (for lac Z).


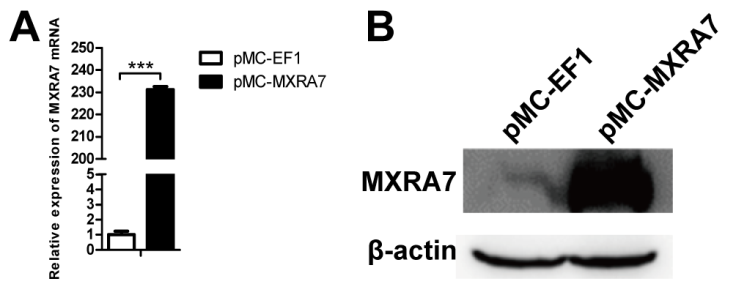


**Figure S2. Construction of MXRA7-delivery plasmid and its expression.** C57BL/6 mice were injected with mock pMC-EF1 (control) or pMC-MXRA7 plasmids (n=6-7 each group) by HGT method. Livers were harvested from the mice four days after injection of plasmids. Expression of MXRA7 in livers was detected by RT-qPCR (A) and western blot (B). ***p<0.001.


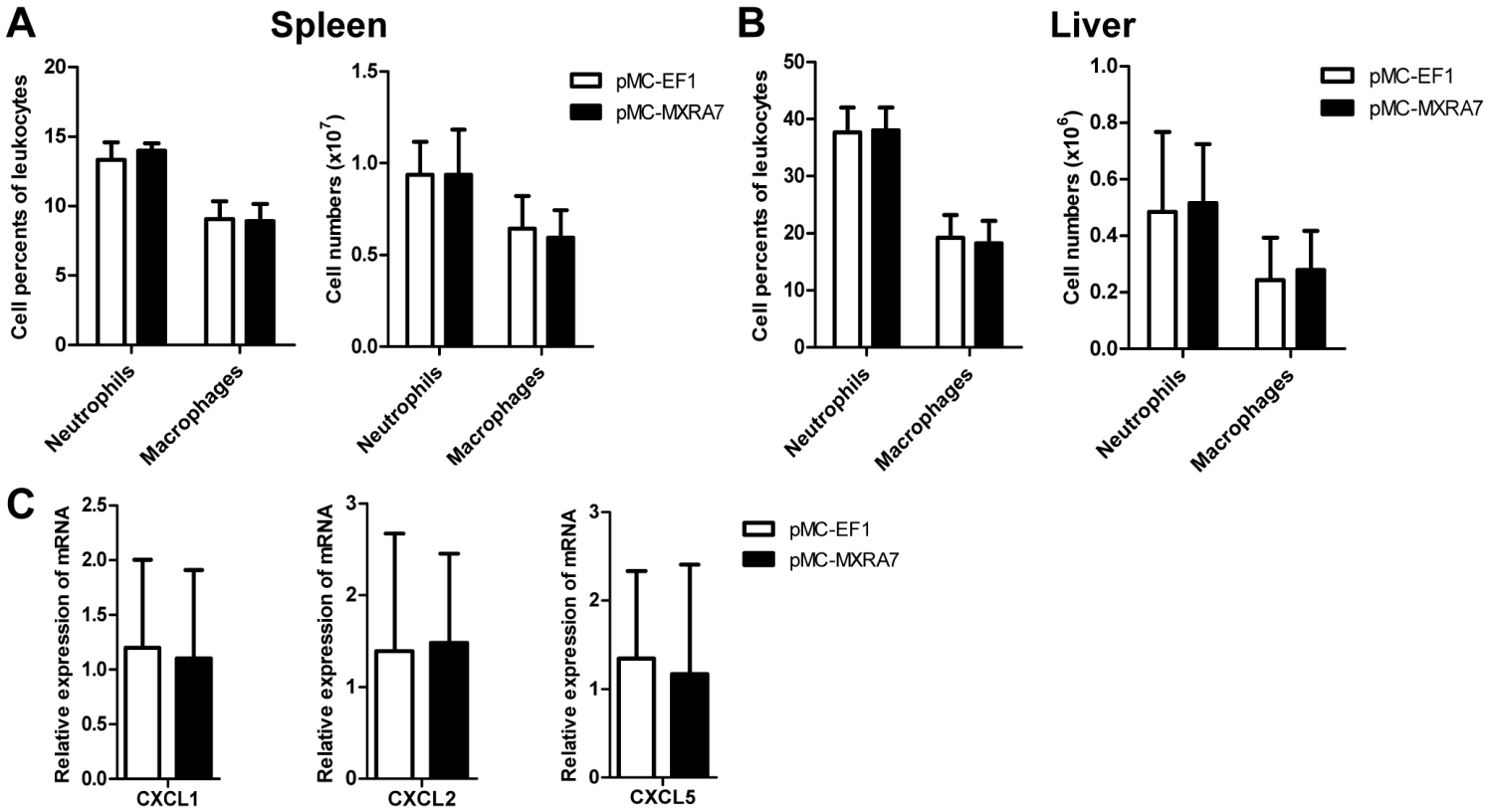


**Figure S3. MXRA7 overexpression has no effect on neutrophils and macrophages infiltration in liver.** (A, B) C57BL/6 mice were injected with mock pMC-EF1 (control) or pMC-MXRA7 plasmids (n=6-7 each group) by HGT method. The mice were sacrificed 24 h after 1ml/kg CCl_4_ injection. Splenocytes and intrahepatic leukocytes were isolated for FACS analysis. Ly6C/Ly6G^+^ neutrophils and F4/80^+^ macrophages in spleen (A) and in liver (B) were measured. (C) Livers were harvested from mice which were sacrificed 24 h after 1ml/kg CCl_4_ injection. Expression of CXCL1, CXCL2 and CXCL5 mRNAs in livers was detected by RT-qPCR.


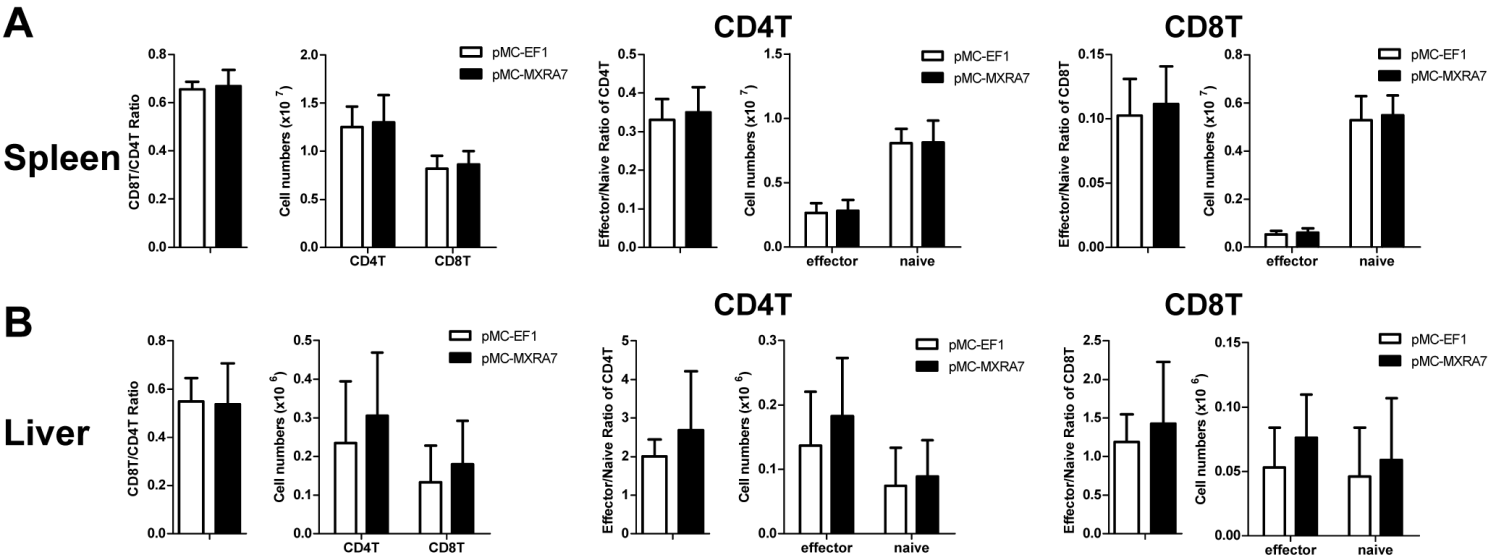


**Figure S4. MXRA7 overexpression has no effect on T cells.** C57BL/6 mice were injected with mock pMC-EF1 (control) or pMC-MXRA7 plasmids (n=6-7 each group) by HGT method. The mice were sacrificed 24 h after 1ml/kg CCl_4_ injection. Splenocytes and intrahepatic leukocytes were isolated for FACS analysis. (A) Flow cytometric analysis of CD4^+^ T and CD8^+^ T cells and effector cells (CD62L^-^CD44^+^) in spleen. (B) Flow cytometric analysis of CD4^+^ T and CD8^+^ T cells, and effector cells (CD62L^-^CD44^+^) in liver. The data shown are the representative of three experiments.


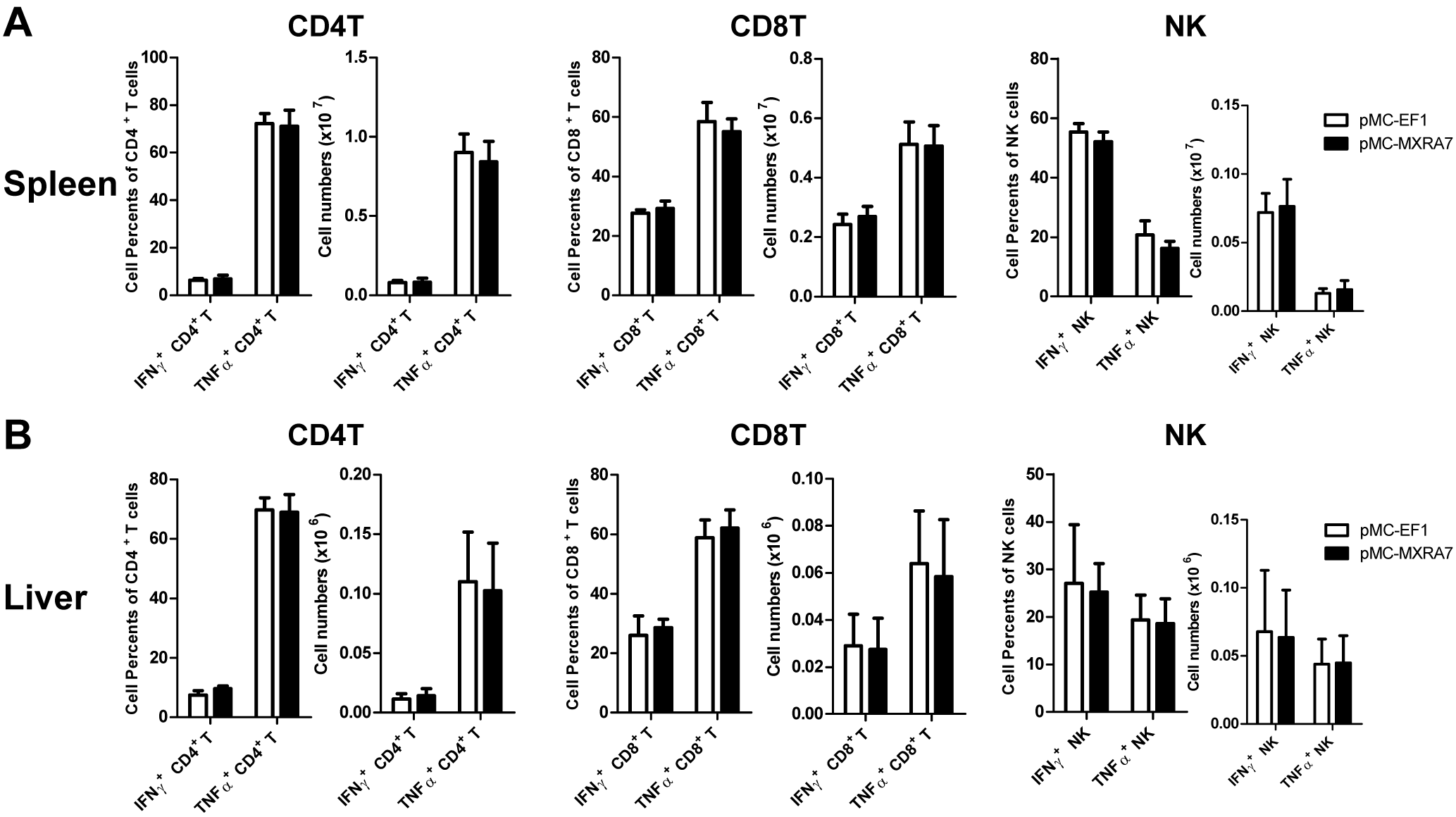


**Figure S5. MXRA7 overexpression has no effect on the production of IFNγ and TNFα from T cells.** C57BL/6 mice were injected with mock pMC-EF1 (control) or pMC-MXRA7 plasmids (n=6-7 each group) by HGT method. The mice were sacrificed 24 h after 1ml/kg CCl_4_ injection. Splenocytes (A) and intrahepatic leukocytes (B) were isolated, stimulated and stained for intracellular cytokines in combination with cell surface staining. The data shown are the representative of three experiments.

**
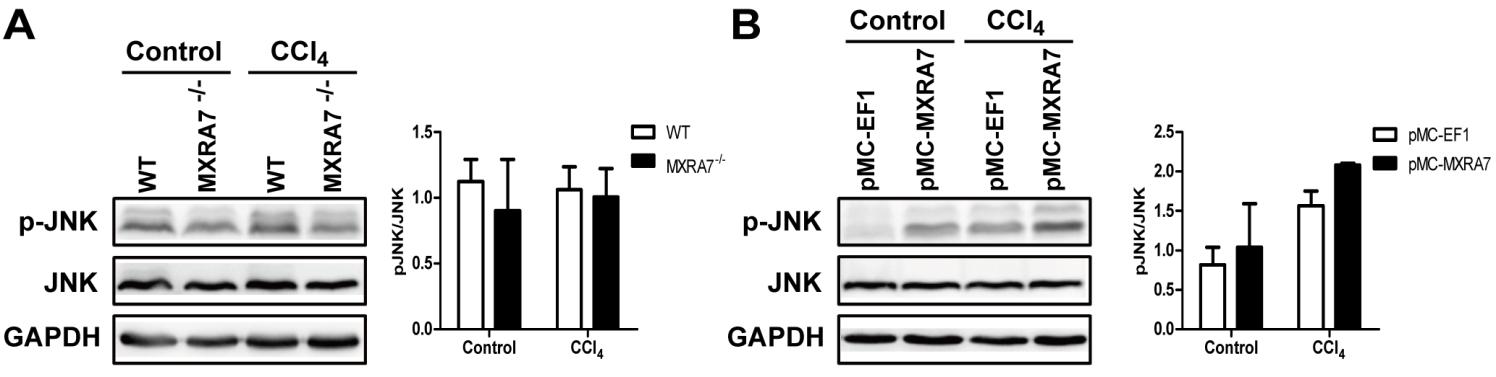
**

**Figure S6. MXRA7 has no effect on the expression of JNK.** Livers were harvested from different groups of mice 24 h after 1ml/kg CCl_4_ injection. (A, B) Proteins were extracted from livers for western blot analysis of p-JNK and JNK, as well as other molecules (refer to Figure 7. Please note that the GAPDH bands were same with those in Figure 7E-F since they were from same batch of assay). Representative images shown are the representative of three independent experiments.
